# Determinants of adherence to recommendations on physical activity, red and processed meat intake, and body weight among lynch syndrome patients

**DOI:** 10.1007/s10689-022-00315-y

**Published:** 2022-09-24

**Authors:** M Hoedjes, A Vrieling, L de Brauwer, A Visser, E Gómez García, N Hoogerbrugge, E Kampman

**Affiliations:** 1 Center of Research on Psychological and Somatic disorders, Department of Medical and Clinical Psychology, Tilburg University, PO Box 90153, 5000 LE Tilburg, The Netherlands; 2 Department for Health Evidence, Radboud university medical center, Nijmegen, The Netherlands; 3 Department of Clinical Genetics, Maastricht University Medical Center, Maastricht, The Netherlands; 4 Division of Human Nutrition and Health, Wageningen University, Wageningen, the Netherlands

**Keywords:** Lynch Syndrome, Cancer prevention, Physical activity, Body weight, Red and processed meat intake, Determinants

## Abstract

This study aimed to identify determinants of adherence to lifestyle and body weight recommendations for cancer prevention among Lynch Syndrome (LS) patients. Cross-sectional baseline data of LS patients participating in the Lifestyle & Lynch (LiLy) study was used to assess determinants of adherence to the World Cancer Research Fund cancer prevention recommendations on body weight, physical activity, and red and processed meat intake. Adherence and potential determinants of adherence were assessed using questionnaires. Multivariable logistic regression analyses were conducted to identify determinants of adherence. Of the 211 participants, 50.2% adhered to the body weight recommendation, 78.7% adhered to the physical activity recommendation, and 33.6% adhered to the red and processed meat recommendation. Being younger and having a higher level of education were associated with adherence to the recommendation on body weight. Having knowledge about the recommendation was associated with adherence to the recommendations on physical activity and red and processed meat. Results confirm that knowledge about recommendations for cancer prevention is an important determinant for adherence and suggest that strategies to increase knowledge should be included in lifestyle promotion targeted at LS patients, along with behavior change techniques influencing other modifiable determinants.

## Introduction

Lynch syndrome (LS) is an inherited cancer syndrome characterized by a high hereditary risk of various cancers, primarily in the colorectum and the endometrium [1]. Worldwide, approximately 28,600 cases of LS are newly diagnosed each year^(2)^. LS is caused by a germline mutation in one of the mismatch repair (MMR) genes [2, 3]. LS patients have a risk of developing colorectal cancer (CRC) between 22 and 69% up to age 70, as opposed to 1–5% in the general Western population [4–6]. Significant differences have been reported in cumulative cancer risk and risk of different cancer types according to MMR gene mutation type (*MLH1, MSH2, MSH6 and PMS2*) [7, 8]. The clinical phenotype of LS has been shown to vary between families, countries, and continents [8], suggesting the importance of the role of environmental and non-genetic factors, such as lifestyle-related factors [9], in the development of cancer [10, 11]. In addition, low penetrance genetic risk factors may be associated with the observed variety in cancer risk among LS patients [12]. The influence of lifestyle-related factors on CRC among LS patients appears to be comparable or even stronger as compared with the general population [11].

Studies investigating the association between lifestyle-related factors and the occurrence of sporadic cancer have shown that lower levels of physical activity and higher body fatness are associated with an increased risk of different types of cancer, including CRC and endometrium cancer [13]. Also, the intake of red and processed meat has been associated with an increased risk of sporadic CRC [13]. Among LS patients, lifestyle-related factors have also been associated with cancer risk. A recent systematic review has shown that early-adulthood overweight/obese weight status and adulthood weight gain may be associated with increased colorectal cancer risk in LS patients [14]. Moreover, a recent meta-analysis has shown that obesity has been associated with an increased risk for colorectal cancer, but only in men with LS [15]. Furthermore, reviews of the current literature among LS patients have shown that high fruit intake and physical activity have been associated with decreased colorectal cancer risk [14], whereas smoking and alcohol consumption have been associated with an increased colorectal cancer risk in LS patients [16].

Based on a large body of scientific evidence for these observed associations, the World Cancer Research Fund (WCRF) has issued lifestyle and body weight recommendations for cancer prevention [13]. Cancer survivors (i.e. those who have been diagnosed with cancer including those who have recovered) are also recommended to meet these lifestyle and body weight recommendations for cancer prevention. Meeting these recommendations has been associated with favorable health-outcomes, such as a higher health-related quality of life, and a decreased risk for type II diabetes mellitus, cardiovascular disease, second primary cancers, cancer recurrences, and mortality [17–20]. Current guidelines from the European Hereditary Tumour Group (EHTG) and European Society of Coloproctology (ESCP) advise health care providers to inform LS patients about the observed associations between lifestyle, body weight and the risk of cancer [16].

We previously found that adherence to WCRF lifestyle and body weight recommendations in LS patients is low and that providing WCRF health promotion materials increased awareness of and knowledge about WCRF recommendations, without increasing psychological distress. However, this did not affect adherence [21]. Little is known on how adherence to these recommendations can best be promoted. Insight into determinants of health behaviors among LS patients is needed to be able to identify what techniques and strategies can be used to achieve health behavior changes in this specific patient population. Apart from our previous qualitative study on determinants of adherence to lifestyle and body weight recommendations among LS patients [22], to our knowledge, no other study has examined determinants of adherence or health behavior change among LS patients. Data on non-changeable determinants associated with (non-)adherence (such as sociodemographic and certain health-related determinants specific to LS, including cancer diagnosis in personal history and years since LS diagnosis) provides insight into which LS patients specifically should be targeted to improve adherence. Data on changeable determinants associated with (non-)adherence provides insight into which modifiable determinants should be targeted for change and informs about what type of techniques or strategies can be used to positively influence these changeable determinants. Such changeable determinants relevant for LS patients include psychological determinants, such as cancer worry and symptoms of depression. These psychological determinants have been associated with unfavorable lifestyle behaviors in previous studies [23, 24]. Besides, behavior change concepts that are frequently included in theories and models of health behavior change are knowledge (about the recommendations) and awareness (of the influence of lifestyle-related factors on cancer risk) [25]. Knowledge and awareness have been shown to be determinants of health behavior in other populations [26, 27].

The aim of this cross-sectional study was to explore demographic, health-related, behavior change and psychological determinants for adherence to body weight, physical activity, and red and processed meat intake recommendations among LS patients, as these specific recommendations are relevant for LS-related types of cancer (CRC, endometrium) [13].

## Methods

### Study design

This study uses cross-sectional, baseline data (n = 218) from the Lifestyle & Lynch (LiLy) study, a randomized controlled trial to test the effect of providing LS patients with WCRF-NL health promotion materials of the WCRF cancer prevention recommendations [21].

### Participants and procedure

The LiLy study recruited participants between April and September 2015 at Radboud University Medical Center and Maastricht University Medical Centre. LS patients aged between 18 and 65 years were eligible for participation if LS diagnoses was confirmed by a germline pathogenic variant in one of the MMR-genes (*MLH1, MSH2, MSH6 or PMS2)*. LS patients were excluded from participation if they had insufficient understanding of the Dutch language or if they were participating in the GeoLynch study, a prospective cohort study among LS patients, to prevent interference between both studies [28]. Since only 4% of eligible LS patients participated in the GeoLynch study, this is unlikely to have biased the results of this study. More information on the LiLy study can be found elsewhere [21].

After informed consent was obtained, eligible LS patients were asked to fill in the baseline questionnaire, which took approximately 45 min to complete. The medical ethical research committees of the Radboud University Medical Center and Maastricht University Medical Centre granted permission to perform this study.

### Measures

#### Adherence to the WCRF recommendations

For this study, adherence to the WCRF recommendations on physical activity, body weight, and red and processed meat intake were included. These recommendations were included as these are relevant for LS-related types of cancer (CRC, endometrium) [13] and the smallest group of each of these dichotomous outcome variables (adherence vs. non-adherence to these recommendations) was large enough to be able to be incorporated into the statistical analyses given the sample size (n = 211)[29].

#### Body weight

Self-reported body weight and height were used to calculated Body Mass Index (BMI) (kg/m^2^). Participants were categorised into the following BMI categories: <18.5; 25-<30;18.5-<25; and > 30 kg/m^2^. The category 18.5–24.9 kg/m^2^ was considered as adherent to the body weight recommendation. The other categories were considered not to be adherent to the body weight recommendation.

#### Physical activity

Adherence to the physical activity recommendation was assessed using the validated Short Questionnaire to Assess Health Enhancing Physical Activity (SQUASH) questionnaire, which contains questions about multiple activities referring to a normal week in the past month. Results were converted to time spent on light, moderate, and vigorous activities, which were then converted to activity scores [30]. When the number of moderate and vigorous exercise activities was at least 30 min a day, for a minimum of 5 days a week, patients were categorized as adherent to the physical activity recommendation.

#### Red and processed meat

Intake of red and processed meat was measured with an adjusted version of a 40-item, validated Food Frequency Questionnaire (FFQ) specifically developed to assess adherence to the Dutch Guidelines for a healthy diet [31]. Items assessing red meat intake (grams per week) and processed meat intake (grams per week) during the last month were used to determine adherence to the recommendation on red and processed meat intake. When red meat intake was < 500 g/w, of which processed meat was < 3 g/d, participants were considered to adhere to the recommendation on red and processed meat intake.

#### Demographic and health-related characteristics

Demographic characteristics were assessed using the baseline questionnaire, which included items on age, gender, marital status, and education. Clinical characteristics were assessed using the same questionnaire by items on personal and family cancer history, colon surgery (colectomy, hemicolectomy, colon resection), time since LS diagnosis, and smoking behaviour.

#### Behavior change and psychological characteristics

##### Awareness

Awareness of the cancer risk factors as described in the WCRF/AICR recommendations for cancer prevention (referred to as awareness of the WCRF/AICR recommendations) was assessed using a question from the AICR Cancer Risk Awareness Survey: “Do the following factors have a significant influence on whether or not the average person develops cancer?”.

From the exposures that were mentioned in the entire Awareness questionnaire reflecting all recommendations, only the exposures related to the recommendations on body weight, insufficient physical activity, and red and processed meat intake were included for the current study. For each exposure, answer options were: “yes, a big influence”; “yes, a small influence”; “no”; and “I do not know”. Participants with correct answers, indicating that the exposures were of influence, were considered to be aware of the specific cancer risk factors while participants with answers “no” and “I do not know“ were considered to be unaware.

##### Knowledge

Knowledge of the WCRF recommendations on body weight, physical activity, and red and processed meat intake was assessed using 3 multiple choice questions; 1 for each recommendation. These knowledge questions required more detailed content-specific knowledge about the recommendations. For example, the multiple choice question “What is the minimally recommended amount of time a day you should be spending on physical activity according to the recommendations for cancer prevention?”, assessed knowledge about the physical activity recommendation. The 5 answer options included: “A recommendation regarding physical activity and cancer risk does not exist”; “A minimum of 30 minutes physical activity per day of moderate intensity (meaning an increased breath and heart rate)”; “A minimum of 60 minutes physical activity per day of moderate intensity”; “A minimum of 90 minutes physical activity per day of moderate intensity”; “I don’t know”. Participants with correct answers were considered to have knowledge about the respective recommendation.

##### Cancer risk perception

Cancer risk perception was assessed by two standardized questions. Participants were asked to express their perceived cancer risk in a percentage between 0 and 100. In addition they were asked to choose one out of 5 categories: ranging from a very low to a very high perceived cancer risk [32].

##### Symptoms of depression

Symptoms of depression were measured by using the Dutch version of the Hospital Anxiety and Depression Scale (HADS) [33]. The HADS consists of 14 items assessing self-reported symptoms of anxiety (7 items) and depression (7 items) in the past week. Each item is scored on a 4-point Likert scale, ranging from 0 to 3, with higher scores indicating more symptoms. For the current study, only scores for symptoms of depression were used (because of the conceptual overlap with cancer worry). A total score can be calculated for symptoms of depression by adding up the scores on the 7 items. This total score ranges from 0 to 21, with higher scores indicating more symptoms [33].

##### Cancer worry

Cancer worry was assessed using the Cancer Worry Scale (CWS), consisting of 8 items. The reliability and validity has shown to be good among breast and colorectal cancer survivors [34, 35]. The total score ranges between 8 and 32, with higher scores corresponding to more cancer worry.

#### Statistical analyses

The population for analysis consisted of participants with complete baseline data. Participants with missing data on one or more of the variables included in the analyses were excluded from the analyses.

Means with standard deviations (SD) and frequency tables were used to describe potential socio-demographic, health-related, and psychological determinants. Since the variables ‘age’ and ‘time since LS diagnosis’ were not normally distributed, these variables were incorporated in the statistical analyses as categorical variables. Age was categorized into the following categories based on the observed data distribution: 21–43 years; 44–54 years; and 55–73 years. Time since LS diagnosis was categorized into the following categories: 0–2 years; 2–4 years; and 4–20 years.

First, univariable logistic regression analyses were conducted with adherence to one of the WCRF recommendations on body weight, physical activity, or red and processed meat intake as dependent dichotomous variable, and a single potential determinant as independent variable. The following potential demographic determinants were included as independent variables: gender (male, female); age (21–43, 44–54, and 55–73 years), education level (low, medium, high), and marital status (partner, no partner). The following potential health-related determinants were included: years since LS diagnosis (0–2 years, 2–4 years, and 4–20 years), colon surgery (yes, no), personal cancer history (yes, no), and smoking status (current, ex-, never smoker). The following potential psychological determinants were included: awareness (yes, no) and knowledge of the recommendations (yes, no), symptoms of depression (continuous), cancer worry (continuous), and cancer risk perception (< 50%, 50%, > 50%).

Subsequently, multivariable logistic regression analyses were conducted with adherence to each recommendation as dependent variable, and as independent variables all socio-demographic, health-related, and psychological characteristics that were found to be statistically significantly associated with adherence in the univariable logistic regression analyses.

Statistical analyses were performed using IBM SPSS Statistics for Windows, version 24. P-values < 0.05 were considered to be statistically significant.

## Results

Of the 218 LS patients who agreed to participate in the study, seven participants with missing data on one or more of the variables were excluded and 211 were included in the population for analysis. Participants were aged between 21 and 73 years (mean 48.2; SD 10.9), and 61.1% was female (N = 129) (Table 1). The number of years since LS diagnosis ranged between 0 and 20 years (mean 3.7; SD 2.7). 18% had had a type of colon surgery (colectomy n = 7, hemicolectomy n = 24, colon resection n = 7).

**Table 1:** Demographic, health-related, behavior change and psychological characteristics in Lynch Syndrome patients (n = 211)

	*Total* *(n = 211)* N (%)*	*Cancer in personal history* *(n = 75)* N(%)*	*No cancer in personal history* *(n = 136)* N(%)*
*Demographic characteristics*			
Gender Female Male	129(61.1) 82(38.9)	45(60.0) 30(40.0)	84(61.8) 52(38.2)
Age at measurement 21–43 years 44–54 years 55–73 years	72(34.1) 74(35.1) 65(30.8)	10(13.3) 34(45.3) 31(41.3)	62(45.6) 40(29.4) 34(25.0)
Educational level			
Low Medium High	20(9.5) 107(50.7) 84(39.8)	7(9.3) 43(57.3) 25(33.3)	13(9.6) 64(47.1) 59(43.4)
Partner Yes No	187(88.6) 24(11.4)	9(12.0) 66(88.0)	15(11.0) 121(89.0)
*Health-related characteristics*			
Years since LS diagnosis 0–2 years 2–4 years 4–20 years	82(38.9) 63(29.9) 66(31.3)	27(36.0) 18(24.0) 30(40.0)	55(40.4) 45(33.1) 36(26.5)
Colon surgery No colon surgery Colon surgery	173(82.0) 38(18.0)	38(50.7) 37(49.3)	135(99.3) 1(0.7)
Smoking status Current smoker Ex-smoker Never smoker	22(10.4) 92(43.6) 97(46.0)	8(10.7) 40(53.3) 27(36.0)	14(10.3) 52(38.2) 70(51.5)
*Behavior change and psychological characteristics*			
Knowledge			
Weight recommendation			
Yes No	111(52.6) 100(47.4)	41(54.7) 34(45.3)	70(51.5) 66(48.5)
Physical activity recommendation Yes No	136(64.5) 75(35.5)	50(66.7) 25(33.3)	86(63.2) 50(36.8)
Meat intake recommendation Yes No	30(14.2) 181(85.8)	10(13.3) 65(86.7)	20(14.7) 116(85.3)
Awareness			
Influence of overweight on cancer risk			
Yes No Influence of physical activity on cancer risk Yes No	154(73.0) 57(27.0) 141(66.8) 70(33.2)	56(74.7) 19(25.3) 52(69.3) 23(30.7)	98(72.1) 38(27.9) 89(65.4) 47(34.6)
Influence of meat intake on cancer risk Yes No	79(37.4) 132(62.6)	35(46.7) 40(53.3)	44(32.4) 92(67.6)
Symptoms of depression [Mean(SD)]	2.78(3.13)	3.4(3.4)	2.4(3.0)
Cancer worry [Mean(SD)]	13.8(4.22)	15.1(4.6)	13.1(3.8)
Cancer risk perception <50% 50% >50%	71(33.6) 51(24.2) 89(42.2)	24(32.0) 16(21.3) 35(46.7)	47(34.6) 35(25.7) 54(39.7)

*Unless otherwise specified; M = mean; SD = standard deviation; BMI = Body Mass Index

The majority of participants were aware of the influence of or had knowledge about the recommendation on body weight (73% and 64.5%, respectively) and physical activity (66.8% and 64.5%, respectively) in relation to cancer risk. Much less participants were aware of the influence of or had knowledge about the recommendation on red and processed meat intake in relation to cancer risk (37.4% and 14,2%, respectively).

Of the 211 participants, 35.5% had a cancer diagnosis in their personal medical history (n = 75), of which 37 had been diagnosed with colorectal cancer, 17 with endometrium cancer, 4 with both colorectal and endometrium cancer, and 17 with other types of cancer. Compared with LS patients without a cancer diagnosis in their personal history, LS patients with a cancer diagnosis were older (*p* < .000), had more often had a type of colon surgery (*p* < .000), were more frequently aware of the influence of meat intake on cancer risk (*p* = .04), and had a higher mean score of depressive symptoms (*p* = .037) and cancer worry (*p* = .001). See Table 1.

### Adherence to the recommendations

Out of the 211 LS patients, 50.2% adhered to the body weight recommendation, 78.7% adhered to the physical activity recommendation, and 33.6% adhered to the red and processed meat intake recommendation.

### Determinants of adherence

#### Body weight recommendation

The univariable logistic regression analyses showed that age 44–54 vs. <44 years, medium and high vs. low educational level, and symptoms of depression were associated with adherence to the body weight recommendation (Table 2).

**Table 2:** Demographic, health-related, behavior change and psychological characteristics of Lynch Syndrome patients (n = 211) and associations with adherence to the WCRF recommendation on body weight [1]

	*Non-adherent* *N = 105*	*Adherent* *N = 106*	*Univariable* ^[[2]]^	*Multivariable* ^[[3]]^
	*N(%)*	*N(%)*	*OR(95%CI)*	*OR(95%CI)*
*Demographic characteristics*				
Gender Female Male	59(56.2) 46(43.8)	70(66.0) 36(34.0)	1.52(0.87–2.65) 1	
Age at measurement 21–43 years 44–54 years 55–73 years	28(26.7) 43(41.0) 34(32.4)	44(41.5) 31(29.2) 31(29.2)	1 0.46(0.24–0.89)* 0.58(0.29–1.14)	1 0.48(0.24–0.94)* 0.86(0.41–1.79)
Education level				
Low Medium High	16(15.2) 56(53.3) 33(31.4)	4(3.8) 51(48.1) 51(48.1)	1 3.64(1.14–11.6)* 6.18(1.90–20.1)**	1 4.55(1.34–15.5)* 6.41(1.83–22.5)**
Partner Yes No	97(92.4) 8(7.6)	90(84.9) 16(15.1)	0.46(0.19–1.14) 1	
*Health-related characteristics*				
Years since LS diagnosis 0–2 years 2–4 years 4–20 years	35(33.3) 33(31.4) 37(35.2)	47(44.3) 30(28.3) 29(27.4)	1 0.68 (0.35–1.31) 0.58(0.30–1.12)	
Colon surgery No surgery Surgery	81(77.1) 24(22.9)	92(86.8) 14(13.2)	1 1.95(0.94–4.02)	
Cancer in personal history				
Yes No	44(41.9) 61(58.1)	31(29.2) 75(70.8)	0.57(0.32–1.01) 1	
Smoking status Current smoker Ex-smoker Never smoker	12(11.4) 54(51.4) 39(37.1)	10(9.4) 38(35.8) 58(54.7)	1 0.84(0.33–2.15) 1.79(0.70–4.53)	
*Behavior change and psychological characteristics*				
Knowledge				
Yes No	55(52.4) 50(47.6)	50(47.2) 56(52.8)	1.02(0.59–1.75) 1	
Awareness				
Yes No	76(72.4) 29(27.6)	78(73.6) 28(26.4)	1.06(0.58–1.95) 1	
Symptoms of depression[Mean(SD)] [4]	3.25(3.22)	2.32(2.98)	0.91(0.83–0.99)*	0.94(0.85–1.03)
Cancer worry [Mean(SD)] [5]	14.4(4.42)	13.3(3.95)	0.94(0.88-1.00)	
Cancer risk perception <50% 50% >50%	39(37.1) 27(25.7) 39(37.1)	32(30.2) 24(22.6) 50(47.2)	1 1.08(0.53–2.23) 1.56(0.83–2.93)	

^*^p < .05, ^**^p < .01, ^***^p < .001

^*1*^ *Body weight recommendation: Body Mass Index 18.5–24.9 kg/m* ^*2*^

^*2*^ *Odds ratios are derived from univariable logistic regression analyses with adherence to the weight recommendation (yes vs. no) as dependent variable and one sociodemographic, health-related or psychological characteristic as independent variable*

^*3*^ *Odds ratios are derived from a multivariable logistic regression analysis with adherence to the weight recommendation (yes vs. no) as dependent variable and all statistically significant (p < .05) sociodemographic, cancer-related, and health-related characteristics in the univariable logistic regression analyses as independent variables*

^*4*^ *Odds ratio per 1 unit increase in the depressive symptoms subscale of the Hospital Anxiety and Depression Scale*

^*5*^ *Odds ratio per 1 unit increase in the Cancer Worry Scale*

In the multivariable analyses, only age 44–54 vs. <44 years (OR 0.48, 95% CI: 0.24–0.94) and medium (OR 4.55, 95% CI: 1.34–15.5) and high (OR 6.41, 95% CI: 1.83–22.5) vs. low educational level remained statistically significantly associated with adherence to the body weight recommendation.

#### Physical activity recommendation

The univariable logistic regression analyses showed that age 55–73 vs. <44 years, ex-smoking vs. current smoking, and having vs. not having knowledge about the physical activity recommendation were associated with adherence to the physical activity recommendation (Table 3).

**Table 3:** Demographic, health-related, behavior change and psychological characteristics of Lynch Syndrome patients (n = 211) and associations with adherence to the WCRF recommendation on physical activity [1]

	*Non-adherent* *N = 45*	*Adherent* *N = 166*	*Univariable* ^[[2]]^	*Multivariable* ^[[3]]^
	*N(%)*	*N(%)*	*OR(95%CI)*	*OR(95%CI)*
*Demographic characteristics*				
Gender Female Male	23(51.1) 22(48.9)	106(63.9) 60(36.1)	1.69(0.87–3.29) 1	
Age at measurement 21–43 years 44–54 years 55–73 years	16(35.6) 23(51.1) 6(13.3)	56(33.7) 51(30.7) 59(35.5)	1 0.63(0.30–1.33) 2.81(1.03–7.69)*	1 0.54(0.25–1.19) 2.44(0.85–6.97)
Education level				
Low Medium High	3(6.7) 22(48.9) 20(44.4)	17(10.2) 85(51.2) 64(38.6)	1 0.68(0.18–2.54) 0.57(0.15–2.13)	
Partner Yes No	42(93.3) 3(6.7)	145(87.3) 21(12.7)	0.49(0.14–1.73) 1	
*Health-related characteristics*				
Years since LS diagnosis 0–2 years 2–4 years 4–20 years	22(48.9) 12(26.7) 11(24.4)	60(36.1) 51(30.7) 55(33.1)	1 1.56(0.70–3.46) 1.83(0.82–4.13)	
Colon surgery No surgery Surgery	33(73.3) 12(26.7)	140(84.3) 26(15.7)	1 1.96(0.90–4.28)	
Cancer in personal history				
Yes No	20(44.4) 25(55.6)	55(33.1) 111(66.9)	0.62(0.32–1.21) 1	
Smoking status Current smoker Ex-smoker Never smoker	8(17.8) 14(31.1) 23(51.1)	14(8.4) 78(47.0) 74(44.6)	1 3.18(1.13-9.00)* 1.84(0.69–4.93)	1 2.59(0.87–7.74) 1.72(0.60–4.95)
*Behavior change and psychological characteristics*				
Knowledge				
Yes No	23(51.1) 22(48.9)	113(68.1) 53(31.9)	2.04(1.04–3.98)* 1	2.22(1.09–4.52)* 1
Awareness				
Yes No	27(60.0) 18(40.0)	114(68.7) 52(31.3)	1.46(0.74–2.89) 1	
Symptoms of depression [Mean(SD)] [4]	3.42(2.86)	2.61(3.19)	0.93(0.84–1.02)	
Cancer worry [Mean(SD)] [5]	13.9 (4.68)	13.8(4.09)	0.99(0.92–1.07)	
Cancer risk perception <50% 50% >50%	14(31.1) 13(28.9) 18(40.0)	57(34.3) 38(22.9) 71(42.8)	1 0.72(0.30–1.70) 0.97(0.44–2.12)	

^*^p < .05, ^**^p < .01, ^***^p < .001

^*1*^ *Physical activity recommendation: moderate to vigorous activities for at least 30 min a day, for a minimum of 5 days a week*

^*2*^ *Odds ratios are derived from univariable logistic regression analyses with adherence to the physical activity recommendation as dependent variable and one sociodemographic, health-related or psychological characteristic as independent variable*

^*3*^ *Odds ratios are derived from a multivariable logistic regression analysis with adherence to the physical activity recommendation (yes vs. no) as dependent variable and all statistically significant (p < .05) sociodemographic, health-related, and psychological characteristics in the univariable logistic regression analyses as independent variables*

^*4*^ *Odds ratio per 1 unit increase in the depressive symptoms subscale of the Hospital Anxiety and Depression Scale*

^*5*^ *Odds ratio per 1 unit increase in the Cancer Worry Scale*

In the multivariable analyses, only having knowledge about the physical activity recommendation remained statistically significantly associated with adherence to this recommendation (OR 2.04, 95% CI: 1.04; 3.98).

#### Red and processed meat intake recommendation

The univariable logistic regression analyses showed that only having vs. not having knowledge about the red and processed meat intake recommendation was associated with adherence to the red and processed meat recommendation (Table 4; OR 2.62, 95% CI: 1.19; 5.74).

**Table 4:** Demographic, health-related, behavior change and psychological characteristics of Lynch Syndrome patients (n = 211) and associations with adherence to the WCRF recommendation on red and processed meat intake [1]

	*Non-adherent* *N = 140*	*Adherent* *N = 71*	*Univariable* ^[[2]]^	*Multivariable* ^[[3]]^
	*N(%)*	*N(%)*	*OR(95%CI)*	*OR(95%CI)*
*Demographic characteristics*				
Gender Female Male	82(58.6) 58(41.4)	47(66.2) 24(33.8)	1.39(0.76–2.51) 1	
Age at measurement 21–43 years 44–54 years 55–73 years	48(34.3) 49(35.0) 43(30.7)	24(33.8) 25(35.2) 22(31.0)	1 1.02(0.51–2.03) 1.02(0.50–2.08)	
Education level				
Low Medium High	14(10.0) 75(53.6) 51(36.4)	6(8.5) 32(45.1) 33(46.5)	1 0.99(0.35–2.82) 1.51(0.53–4.32)	
Partner Yes No	121(86.4) 19(13.6)	66(93.0) 5(7.0)	2.07(0.74–5.81) 1	
*Health-related characteristics*				
Years since LS diagnosis 0–2 years 2–4 years 4–20 years	58(41.4) 38(27.1) 44(31.4)	24(33.8) 25(35.2) 22(31.0)	1 1.59(0.80–3.18) 1.21(0.60–2.43)	
Colon surgery No surgery Surgery	111(79.3) 29(20.7)	62(87.3) 9(12.7)	1 1.80(0.80–4.04)	
Cancer in personal history				
Yes No	55(39.3) 85(60.7)	20(28.2) 51(71.8)	0.61(0.33–1.13) 1	
Smoking status Current smoker Ex-smoker Never smoker	19(13.6) 59(42.1) 62(44.3)	3(4.2) 33(46.5) 35(49.3)	1 3.54(0.98–12.9) 3.58(0.99–12.9)	
*Behavior change and psychological characteristics*				
Knowledge				
Yes No	14(10.0) 126(90.0)	16(22.5) 55(77.5)	2.62(1.19–5.74)* 1	2.62(1.19–5.74)* 1
Awareness				
Yes No	50(35.7) 90(64.3)	29(40.8) 42(59.2)	1.24(0.69–2.23) 1	
Symptoms of depression [Mean(SD)] [4]	2.87(3.13)	2.61(3.16)	0.97(0.89–1.07)	
Cancer worry [Mean(SD)] [5]	14.1(4.52)	13.2(3.48)	0.94(0.88–1.01)	
Cancer risk perception <50% 50% >50%	46(32.9) 32(22.9) 62(44.3)	25(35.5) 19(26.8) 27(38.0)	1 1.09(0.52–2.31) 0.80(0.41–1.56)	

^*^p < .05, ^**^p < .01, ^***^p < .001

^*1*^ *Meat intake recommendation: <500 g/w red meat, < 3 g/d processed meat*

^*2*^ *Odds ratios are derived from univariable logistic regression analyses with adherence to the WCRF red and processed meat intake recommendation (yes vs. no) as dependent variable and one sociodemographic, health-related or psychological characteristic as independent variable*

^*3*^ *The independent variable Knowledge is the only variable that was statistically significantly (p < .05) associated with adherence to the WCRF red and processed meat intake recommendation (yes vs. no) in the univariable logistic regression analyses*

^*4*^ *Odds ratio per 1 unit increase in the depressive symptoms subscale of the Hospital Anxiety and Depression Scale*

^*5*^ *Odds ratio per 1 unit increase in the Cancer Worry Scale*

## Discussion

This first quantitative explorative study on determinants of adherence to WCRF lifestyle and body weight recommendations for cancer prevention in LS patients showed that knowledge about the recommendations was a statistically significant determinant of adherence to the lifestyle recommendations on physical activity and red and processed meat intake. Being younger and having a higher level of education were associated with adherence to the recommendation on body weight.

Adherence to the body weight recommendation among LS patients in the current study was comparable to adherence in the general Dutch population in which 50% of those aged 18 and older adhered to the body weight recommendation [36]. As compared to an observational study in Dutch colorectal cancer survivors, adherence to the recommendations on body weight (50% vs. 34%), physical activity (78.7% vs. 73%), and red and processed meat (33.6% vs. 8%) was higher in the LS patients participating in the current study [37].

To our knowledge, no other studies have quantitatively investigated determinants of adherence to lifestyle and body weight recommendations in LS patients. The results of this first quantitative exploration of determinants of adherence are in accordance with our previous qualitative findings showing that having knowledge about the recommendations serves as a cue to action for adherence to lifestyle recommendations in LS patients [22]. Knowledge is incorporated as a determinant in multiple frequently used theories and models of health behavior change (e.g., the theory of planned behavior, the Health Belief Model, Social Cognitive Theory) [25]. In this study, knowledge was found to be a determinant of adherence to the recommendations on health behaviors (physical activity and red and processed meat intake), but not of adherence to the body weight recommendation. These findings may be explained by the theoretical proximity of the determinant knowledge to a certain health behavior (such as physical activity or red and processed meat intake) as opposed to an outcome of multiple lifestyle behaviors (body weight). Considering that adherence to the body weight recommendation is subject to adherence to recommendations on energy balancing behaviors (physical activity, sedentary behavior, and dietary intake), it seems plausible that knowledge is a more proximal determinant of health behaviors and a more distal determinant of adherence to the body weight recommendation (outcome of the health behaviors physical activity and diet quality). In other words, it makes sense that it’s more difficult to influence (the result of) multiple lifestyle behaviors just by increasing knowledge than it is to influence a single lifestyle behavior. Hence, this could explain our finding that knowledge was found to be a statistically significant determinant of the health behaviors physical activity and red and processed meat intake, but not for the outcome of health behaviors (body weight).

The observed association between adherence to the body weight recommendation and educational level is in line with previous research. A large Canadian cross-sectional study examining determinants of adherence to WCRF recommendations in the general population, also found that higher education attainment was associated with higher odds of adhering to the recommendation for body weight [38].

It should be noted that most of the potential determinants of adherence included in this study did not show a statistically significant association with adherence to recommendations on body weight, physical activity, and red and processed meat intake. Contrary to our expectations, having a cancer diagnosis in one’s personal medical history was not found to be statistically significantly associated with adherence. This seems to be in disagreement with the presumed window of opportunity for lifestyle change after a cancer diagnosis that has been described in the scientific literature on health behavior change after a cancer diagnosis [39]. In addition, time after LS diagnosis also was not found to be statistically significantly associated with adherence.

### Strengths and limitations

A strength of this first quantitative study on determinants of adherence to WCRF lifestyle recommendations for cancer prevention in LS patients is the relatively large sample size (n = 211) in relation to the number of LS patients (estimated 10-year prevalence of 3.316 in the Netherlands) [40, 41]. Other strengths include the extensive assessment of adherence to the recommendations and potential determinants and the use of widely-used validated questionnaires.

Several limitations should be considered when interpreting the results of this study. Our study sample consisted of LS patients who agreed to participate in a study about lifestyle and cancer risk (response rate 53%). LS patients who participated were more likely to be older, female, and to have had a previous diagnosis of cancer compared with those who did not participate. Therefore, our study sample may not be a representative sample of LS patients. In addition, our sample consisted of a relatively high proportion of highly educated individuals, which may limit the generalizability of our findings and may reflect an overestimation of the proportion of LS patients having knowledge about the recommendations. Furthermore, while interpreting our findings, it should be taken into account that adherence to lifestyle and body weight recommendations was assessed using self-report questionnaires, which may have led to over-reporting of healthy lifestyle behavior and under-reporting of body weight, particularly among individuals with overweight or obesity [42, 43]. Additionally, the sample size (n = 211) was too small to be able to enter all independent variables into one multivariable logistic regression analyses as the validated rule of thumb of a minimum of 10 participants per independent categorical variable in the smallest group would have been violated [44]. Therefore, only the independent variables that were statistically significantly associated with adherence were entered into the multivariable logistic regression analyses. It should also be noted that we did not distinguish between different MMR genes in our statistical analyses, while the cumulative cancer risk and the risk of different cancer types differs according to MMR gene mutation type [7]. Since we found that having been diagnosed with (any type of) cancer was not associated with adherence this is not expected to influence our results. Finally, it should be noted that there are many more possible determinants of health behavior change that we did not incorporate in this study that may have influenced adherence. Such possible determinants include for example social and environmental factors, which should be incorporated in future studies to provide a more comprehensive picture of the determinants of adherence to lifestyle recommendations in LS patients.

The results of this study confirm the importance of having knowledge about lifestyle recommendations and suggest that such knowledge should be promoted to achieve adherence. Our previous publication about the LiLy study has shown that knowledge about lifestyle recommendations can be increased by providing LS patients with WCRF-NL health promotion materials [21]. Health care providers involved in (follow-up) care for LS patients (such as genetic counsellors, clinical geneticists, gastro-enterologists, gynaecologists) could easily incorporate providing WCRF-NL health promotion materials during counselling or surveillance visits with LS patients. Informing LS patients about lifestyle-related factors (including the preventive use of aspirin [45]) and cancer risk is in line with current guidelines for LS patients [16]. Increasing knowledge, by providing health promotion materials or referring to online health education material (e.g., via the international and national websites of the WCRF such as www.wcrf.org), is an important first step to achieve adherence. When health care professionals provide these materials, this is in itself an additional behavior change technique (credible source) [46]. However, as our previous study and many others have shown, health behavior change is not likely to be achieved by solely providing information [21, 47]. Although information provision is an important first step towards health behavior change, typically, a combination of multiple behavior change techniques and strategies targeting a multitude of health behavior determinants is needed to achieve and maintain health behavior changes. Therefore, health care professionals treating LS patients could refer to other health care professionals specialized in health behavior change (such as a dietician, physical therapist, or a lifestyle coach). They could provide these additional behavior change techniques to achieve health behavior changes and to improve health outcomes in LS patients.

## Conclusion

The results of this first quantitative study on determinants of adherence to WCRF lifestyle and body weight recommendations among LS patients confirm that knowledge about the recommendation is an important determinant for adherence to the recommendations on physical activity and red and processed meat intake. Results suggest that strategies to increase knowledge (e.g., providing health education materials) should be included in lifestyle promotion targeted at LS patients, along with behavior change techniques influencing other modifiable determinants of adherence.

## Data Availability

Data is available upon request from the corresponding author.
